# The Impact of Different Uses of the Internet on Farmers′ Adoption of Soil Testing and Formulated Fertilization Technology in Rural China

**DOI:** 10.3390/ijerph20010562

**Published:** 2022-12-29

**Authors:** Junxia Zeng, Dengwang Li, Cuiping Ma, Bin Wang, Liangliang Gao

**Affiliations:** Institute of Rural Development at Chinese Academy of Social Sciences, Beijing 100732, China

**Keywords:** green agriculture, Internet use, soil testing, formulated fertilization, China

## Abstract

Soil testing and formulated fertilization technology can effectively solve the problem of the excessive and inefficient use of chemical fertilizers. Previous studies have found that the use of the Internet can increase the adoption of soil testing and formulated fertilization technology among farmers. However, they do not distinguish between the effects of the different uses of the Internet (with or without productive use) on the adoption of soil testing and formulated fertilization technology. This study investigates the Internet use of 5341 professional farmers in rural China in 2019, finding that 18.97% of them still use the Internet for only communication and entertainment and do not use any agricultural productive services on the Internet. The adoption rate of soil testing and fertilization technology among these farmers is only 23.77%, which is approximately 10 percentage points lower than that of farmers who use the Internet for productive purposes. The double robust model shows that the probability of the adoption of soil testing and formulated fertilization technology by farmers with productive use of the Internet increases by six percentage points, which is both statistically and economically significant. In the future, China should train more farmers to use the Internet for productive purposes; this will help more farmers, particularly those with low skills and low educational attainment, to use the Internet and play a positive role in promoting the Internet for green agricultural production techniques.

## 1. Introduction

Agricultural green transformation and sustainable development are important elements of the UN Sustainable Development Goals and the transformation of agri-food systems. Fertilizer use is an important indicator of green agricultural development. China is the largest fertilizer user worldwide [1]. Fertilizer application intensity has increased from 265 kg/hm^2^ in 2000 to 313 kg/hm^2^ in 2020, which is significantly higher than the international safety standard of 225 kg/hm^2^. Furthermore, the fertilizer utilization rate with the three major food crops, rice, maize, and wheat, was only 39.2% in 2019 [2]. Fertilizer pollution in Chinese agriculture is significantly severe, and excessive fertilizer application is very common, particularly among smallholder farmers [3]. Since 2015, the Chinese government has introduced a series of policies to reduce the use of chemical fertilizers [4], one of which is soil testing and formulated fertilization technology.

Soil testing and formulated fertilization technology comprise environmentally friendly technologies promoted by the United Nations and supported by the Chinese government through financial subsidies since 2005. Soil and field fertilizer testing can effectively avoid excessive inputs of nitrogen, phosphorus, potassium, and other elements by formulating fertilizers according to crop categories and soil conditions. Through soil testing and formulated fertilization technology, the utilization rates of nitrogen, phosphorus, and potassium fertilizers for the three major food crops in China were 33%, 24%, and 42% in 2015, which are increases of 5, 12, and 10 percentage points, respectively, compared to the pre-project period in 2005 [5]. However, the adoption rate of soil testing and formulated fertilization technology among Chinese farmers is less than one-third, which is significantly lower than those in developed countries.

Farmers are the main operating entities of agricultural production and operation, and many scholars have conducted rich discussions on the behavior and influencing factors of the adoption of soil testing and formulated fertilization technology (including many agricultural green production technologies) by farmers. The factors influencing the adoption of green production technologies are three categories: One is the individual characteristics of farmers, such as gender, age [6], educational level [7], technical training [3,8,9,10], awareness of environmental regulations, and experience in agricultural production [11,12,13]. Another is the household characteristics of farmers, including household size [14], household income, part-time employment, and non-farm labor [15,16,17,18]. The third one is the production and operation characteristics, including farming experience [19], land operation scale [20], land fragmentation [21], land right stability [22], land ownership [23], land transfer [24], cooperative membership [7,25], extension contact [26], and peer effects [27].

Some scholars have found that Internet use has increased fertilizer expenditures [28] and the adoption of green production technologies, such as soil testing and formulated fertilization [29,30]. However, existing literature does not distinguish between Internet uses (e.g., use for production and use for life), ignoring the fact that the different uses of the Internet may have different impacts on the adoption of green technologies by farmers. Some farmers use the Internet only for communication and entertainment functions but not for some productive services in agriculture because of their low-human capital level and inadequate Internet use skills. The Internet belongs to skill-biased technical change: the higher the educational attainment and skills of workers, the higher the proportion of using the Internet for study and work and the stronger the Internet′s contribution to their labor productivity and employment rate. However, the lower the educational attainment and skills of workers, the longer they use the Internet for leisure and entertainment and the weaker the promotion effect on their labor productivity and employment rate [31], and this may have a significantly negative impact [32,33].

This study examines the impact of different uses of the Internet on the adoption of soil testing and formulated fertilization technology by Chinese farmers. This study divides Internet use into two categories according to the presence or absence of productive agricultural use. By using data from 5341 professional farmers in China in 2019, we find that the adoption rate of soil testing and formulated fertilization technology in the total sample of farmers is 33.36%. However, farmers with productive Internet use have an approximately 10-percentage point higher adoption rate than those without productive use. Results from double robust models show that productive use of the Internet can lead to at least a 5.92 percentage point increase in the adoption of soil testing and formulated fertilization, which is both statistically and economically significant.

The contributions of this study are two-fold: First, it differentiates Internet use and examines the different effects of the different uses on the adoption of soil testing and formulated fertilization technologies by farmers. Second, the Internet use and adoption of green production technologies among professional Chinese farmers are studied. Professional Chinese farmers are different from small farmers in general because they have specific skills, are engaged in large-scale agricultural production and operation activities, and are more market-oriented [34]. However, the percentage of those with a junior high school education or below is 49% in 2020 [35], which suggests that the education level of professional Chinese farmers is still low. The low level of education may prevent professional farmers from benefiting from Internet use because of low skill constraints, which is found in this study.

The remainder of this paper is organized as follows: Section 2 describes the data, methods, and variables of the study. Section 3 presents the empirical findings and mechanism analysis to explore the impact of the productive use of the Internet on the adoption of soil testing and formulated fertilization technology by farmers. Section 4 presents conclusions and possible policy recommendations.

## 2. Data and Methods

### 2.1. Data

The data used in this study were obtained from the 2019 Farmer Quality Development Tracking Survey organized by the Central Agricultural Broadcasting and Television School (Farmer Science and Technology Education and Training Center of the Ministry of Agriculture and Rural Affairs). In 2019, the number of professional farmers in China reached 16 million, accounting for 8.23% of China′s agricultural labor force. This survey has the largest sample size and was the most representative national survey of professional farmers. In 2018, the survey questionnaire collected information on basic personal and family information, education and training, agricultural production and operation, product sales, and cooperative production of professional farmers. The survey also covers farmers’ use of the Internet through smartphones, computers, or tablets. This survey provides a solid database for studying the impact of different Internet uses by professional farmers on their adoption of green production technologies.

The survey was conducted in 30 provinces, cities, and autonomous regions of mainland China (excluding Tibet), with the following sampling principles: Within each province (city and region), all counties were divided into three tiers of high, medium, and low according to the gross domestic product (GDP) per capita ranking of each county, and two counties (or districts) were randomly selected from each tier. Thus, six counties (or districts) were selected in each province. Within each county (or district), all townships were divided into three tiers according to the per capita disposable income of farmers, and two townships were randomly selected in each tier, for a total of six townships. Ten farmers were randomly surveyed in each township.

In total, 9227 farmers from 168 counties and districts in 30 provinces of mainland China participated in this survey, of which 6404 were certified as professional farmers by local agricultural departments. Given that this study adopts soil testing and formulated fertilization technology, 664 farmers engaged in only animal husbandry were excluded. After excluding those with missing variables, the final sample included in the analysis was 5341 professional farmers who had obtained a professional farmer certificate and were engaged in planting or combined planting and animal husbandry.

### 2.2. Methodology

#### 2.2.1. Regression Model

To estimate the effect of different Internet uses on the adoption of soil testing and formulated fertilization technology by farmers, the following model was constructed:

*y_i_* = *α*_0_ + *α*_1_ × *production_use_i_* + *α*_2_ × *X_i_* + *ε_i_* (1)

In Equation (1), the dependent variable *y_i_* is a dummy variable that indicates if the *i* respondent used soil testing and formulated fertilization technology (yes = 1, no = 0). Considering that the dependent variable was a dummy variable, the econometric regression models used in this study are Logit, Probit, and linear probability (LPM) models.

The core independent variable *production_use_i_* is a dummy variable that indicates if there is productive use of the Internet by individuals (yes = 1, no = 0). *X_i_* is a control variable that mainly includes the individual characteristics of respondents, i.e., gender, age, the square of age, education level, whether they are technically skilled or not (whether a professional farmer has obtained any farmer technical certificate or professional qualification certificate), if they have participated in agricultural training, and if they are village cadres. Furthermore, it includes household characteristics, such as the type of household entry road, number of household members, and number of household agricultural laborers. Furthermore, it includes agricultural production and operation characteristics, such as the total area of land operation, type of production and operation, main crops, whether they are family farms or not, if they participate in farmers′ cooperatives, and if they have cooperation with agricultural companies. Additionally, this study controlled for province-fixed effects.

#### 2.2.2. Double Robust Model

The group of farmers with productive Internet use may be selective, perhaps with specific characteristics that influence both the productive service functions of the Internet used by farmers and their adoption of soil testing and formulated fertilization technology. However, it is difficult to observe and measure these characteristics. Therefore, estimations using Logit, Probit, or LPM regression models may suffer from endogeneity problems owing to sample selectivity bias. In this study, the endogeneity problem was addressed using inverse probability weighted regression adjustment (IPWRA) and augmented inverse probability weighted (AIPW) treatment effect models. The sample of professional farmers with productive Internet use was considered the treatment group, and the sample of professional farmers without productive use was considered the control group. The selection model and outcome model are constructed as follows:

production_use*_i_* = *β*_0_ + *β*_1_ × *Z_i_* + *u_i_* (2)

*y_i_* = *δ*_0_ + *δ*_1_ × *W_i_* + *v_i_* (3)

Equation (2) is the selection model (or Internet use equation), which is used to estimate the probability of productive Internet use by individuals, and Equation (3) is the outcome model (or the soil testing and formulated fertilization technology adoption equation), which is used to estimate if an individual adopts soil testing technology. The selection model can be estimated by the Logit model. The outcome model can be estimated by Logit, Probit, or LPM models. *Z_i_* in the Internet use equation denotes the variables that may influence individual productive Internet use; *β*_1_ is its estimated coefficient. *W_i_* in the green technology adoption equation denotes the variables that may influence the individual adoption of soil testing and formulated fertilization technology; *δ*_1_ is its estimated coefficient.

As suggested in the literature [36,37,38,39], The advantage of IPWRA and AIPW models is that they are doubly robust. Both models are a combination of regression adjustment (RA) and inverse probability weighting (IPW) estimation methods. Both models significantly mitigated the endogeneity problem by correcting the estimation results of the outcome model using the estimation results of the selection model. IPWRA and AIPW models do not require both the outcome and selection models to be accurately set. As long as one of the two settings is accurate, the estimation results are robust, and thus, they are doubly robust. Additionally, the estimation error was relatively small, even if the settings of both the outcome and selection models were biased.

In the IPWRA model, the first step is to estimate the Internet use equation and calculate the inverse probability weights. The second step is to use the inverse probability weights calculated in the first step to estimate the adoption equation for soil testing and formulated fertilization technology and calculate the predicted irrigation rate of the individual adoption of soil testing technology. The third step is to calculate the mean probability of the adoption of soil testing and formulated fertilization technology for different treatment groups (productive Internet use group and no productive use group). The first step in the AIPW model is the same as that in the IPWAR model; in the second step, the adoption equation is estimated without using the inverse probability weights calculated in the first step. In the third step, the adoption equation is estimated using the inverse probability weights calculated in the first step. Further, the inverse probability weights calculated in the first step are used to weigh the estimated predicted probabilities of adoption of soil testing and formulated fertilization technology for different treatment groups (productive use of the Internet and unproductive use). Furthermore, the difference between the mean of predicted probabilities of the two groups is the effect of the productive use of the Internet on the adoption of soil testing and formulated fertilization technology [40].

For the selection model, two instrument variables (IV) were used, including the mean value of the productive agricultural use of the Internet by farmers in the respondent′s district and county and if the household has broadband access. These two variables affect Internet use directly, but do not affect the adoption of soil testing and formulated fertilization technology. All the control variables used in the selection model are the same as those in Equation (1). All the control variables used in the outcome model are also the same as those in Equation (1).

### 2.3. Variable Description

#### 2.3.1. Outcome Variable

This study determined if farmers use soil testing and formulated fertilization technology, measured by “1 = yes, 0 = no”. We understand that there are some farmers who did not carry out the formula for soil testing. There are also some farmers who carry out the formula for soil testing but do not apply fertilizer according to the results. In this paper, we define those who both carry out the formula for soil testing and apply fertilizer according to the results as “the adoption of soil testing and formulated fertilization technology”. In the total sample, only 31.54% of professional farmers used soil testing and formulated fertilization technology, indicating that the proportion of professional farmers using soil testing and formulation is still relatively low.

#### 2.3.2. Explanatory Variables

The core explanatory variable in this study was the different uses of the Internet, which were divided into two categories based on whether or not farmers use the Internet for information service functions that contribute to agricultural production and operation. The first category was unproductive use, and the second category was productive use. The unproductive uses mainly include the communication functions of the Internet (such as making phone calls and chatting with WeChat, which has become the preferred means of communication for Chinese people) and the leisure and entertainment functions (including using the Internet to watch entertainment videos, news, and games). The productive uses (related to agriculture) include learning functions (learning knowledge of agricultural production and management), information acquisition (obtaining product market information), and e-commerce (purchasing agricultural materials and selling agricultural products online). If a farmer does not use any of the productive agricultural functions and uses only the communication and entertainment functions, it is classified in the first category; if the farmer uses any of the productive agricultural functions, it is classified in the second category.

Table 1 shows that 18.97% of farmers use the Internet unproductively, and 81.03% use it productively. Among different Internet applications, 95.17% used Internet devices to make phone calls and chat with WeChat; 45.98% used the Internet for leisure and entertainment; 65.12% used it to learn about agricultural production and management; 64.33% used it to inquire about and obtain product market information; 47.78% used it for the e-commerce of agricultural products. In short, of professional farmers who were more educated and professional in agricultural production, nearly one-fifth of them used Internet devices only for communication and entertainment but not for any productive service use.

#### 2.3.3. Control Variables

According to existing literature, farmers′ characteristics, family characteristics, and production and business characteristics are important factors that determine if farmers adopt green production technologies. Based on data availability, the influencing factors that affect the adoption of green technologies in agriculture by farmers were selected as control variables. They included personal characteristics (gender, age, education level, whether or not they were technically skilled, whether or not they were village cadres, whether or not they had participated in agricultural production and management training) [7,9] and household characteristics (type of household road, household size, household farming population) [16,17]. Furthermore, they included production and management characteristics (total land operation area, type of production and management, most important crop type, whether or not it is a family farm, whether or not it is a member of a cooperative, and whether or not it cooperates with an agricultural company) [24,41,42]. Additionally, considering the regional differences in geography, economy, and policies in different provinces, provincial dummies were also controlled. Table 2 presents the definitions and descriptive statistics for each variable.

## 3. Results

### 3.1. Descriptive Analysis

Considering the different uses of the Internet, only 23.4% of farmers without productive use used soil testing technology, while 33.36% of farmers with productive use used soil testing technology, which was 9.96 percentage points higher than the former. The descriptive statistics showed that there was a significant difference between the two, and econometric models will be used later to control for other possible variables to see if a significant difference still exists between the two.

Furthermore, there were significant differences in some control variables between the two categories of farmers with and without productive Internet use (Table 2). For example, compared with farmers without productive use, farmers with productive use were 2.89 years younger, 15.62 percentage points more likely to have a high school educational attainment or above, and 11.18 percentage points more likely to have a concrete or asphalt road in the household. Additionally, farmers with productive use of the Internet have 48.3 mu more land than those without productive use of the Internet, approximately 1.5 times larger than their counterparts. Farmers with productive use of the Internet are more organized in production, with a higher percentage of family farms, a higher percentage of cooperatives, and a higher percentage of cooperation with agricultural enterprises. The proportions of family farms, cooperatives, and agricultural enterprises were all more organized. This suggests that there may be some systematic differences between farmers with and without the productive use of the Internet, and possible endogeneity should be considered in the regression analysis.

### 3.2. Regression Results

Whether farmers adopted soil testing and formulated fertilization technology was a dummy variable; thus, the Logit model (regression 1 in Table 3), Probit model (regression 2 in Table 3), and LPM model (regression 3 in Table 3) were used to calculate the impact of the different uses of the Internet.

The three models shown in Table 3, after controlling for individual characteristics, household characteristics, production and business characteristics, and province fixed effects, indicate that farmers with the productive use of the Internet have a significantly higher probability of adopting soil testing technology than farmers without any productive use. The magnitude and significance of the three models were very consistent, with a marginal effect of 0.046 for the Logit model and the Probit model, indicating that the farmers with the productive use of the Internet for the adoption of soil testing increased by 4.6 percentage points, which was significant at the 10% level. The marginal effect of productive use of the Internet in LPM model is smaller than those in the Logit or Probit models, but it is still significant at the 10% level.

Among the control variables, male farmers have a relatively high adoption rate of soil testing and formulated fertilization technology techniques, which may be due to better knowledge of chemical inputs and health risks compared with female farmers [7]. The adoption rate is relatively high among better-educated farmers because soil fertilizer testing is a knowledge-intensive production factor. Farmers with higher education levels are more likely to reduce the intensity of chemical fertilizers and pesticides [9].

The cost reduction and benefits increase from soil testing and formulated fertilization technology were related to the scale of land operation. Within a specific scale, the probability of farmers adopting soil testing and fertilizer increase as the scale area of land operation increase. The probability of adopting soil testing and formulated fertilization technology increase with an increase in land operation scale. Compared with general farmers, family farmers have a relatively strong awareness and behavior toward ecological protection and green production.

Furthermore, the adoption rate of soil testing and formulated fertilization technology is relatively low among farmers with a relatively large number of agricultural laborers. This may be because soil testing and formulated fertilization technology promote the rational use of agricultural production factors and are generally more labor-saving. Owing to the diversification of the operation, the adoption rate of soil testing and formulated fertilization technology in the type of combined planting and animal husbandry is lower than that of the pure farming type. Additionally, crop type affects the adoption rate of soil testing and formulated fertilization technology; for example, the adoption rates of farmers who grow corn and fruits were lower than those of wheat.

### 3.3. Double Robust Estimation

Whether farmers adopt soil testing technology or not is influenced by various factors, some of which are unobservable or measurable; thus, there may be a sample self-selection problem that makes the estimation results biased. Considering the possible endogeneity problem, this study uses a combination of two treatment effect models, IPWRA and AIPW, to mitigate the endogeneity problem. To obtain more accurate standard errors when using the above two models, the standard errors were estimated by 1000 replications using Bootstrap methods.

Table 4 presents the result of the selection model for the productive use of the Internet. Results show that the mean value of the productive Internet use among farmers in the districts and counties of farmers and broadband access to households have a positive effect on productive use of the Internet. Males, educational level, village cadres, cement or asphalt household roads, land operation scale, type of farming combination, and vegetable and fruit crops are also positively related to the productive use of the Internet. The productive use of the Internet is biased toward farmers with more human capital, social capital, and physical capital and farmers with relatively high technical requirements for producing cultivated crops (e.g., vegetables, and fruits). Farmers with technical skill designations have significantly less productive Internet use, probably because they already have some knowledge and skills in agricultural production and management and do not need to resort to the Internet. This indirectly indicates the need for training on Internet use for farmers with relatively few skills.

Table 5 gives the predicted probability of adoption of soil testing and formulated fertilization technology from double robust models. The results of both the IPWRA and AIPW models indicate that farmers with productive Internet use have a significantly higher probability of adopting soil testing and formulated fertilization technology than farmers without productive use. The estimation of average treatment effects (ATE) indicates that the productive use of the Internet has a significant positive impact on the adoption of soil testing and formulated fertilization technologies by farmers, and this impact was robust (see Table 5). Comparing against the result in Table 3, the magnitude of the impact of productive use of the Internet is larger than that in Table 3. Given that 33.36% of farmers in the total sample used soil testing and formulated fertilization technology, the productive use of the Internet led to an 18.1% (6.05/33.36)~18.9% (6.32/33.36) increase in the adoption of soil testing and formulated fertilization technology by farmers, which is highly economically significant.

### 3.4. Potential Mechanism Analysis

How has the productive use of the Internet facilitated the adoption of soil testing and formulated fertilization technology by farmers compared with the unproductive use of the Internet? It can be understood in several ways that Internet use improves the human capital, social capital, and economic capital of farmers.

First, the Internet is an efficient, real-time, and low-cost information access channel through which farmers can attain agricultural production and management knowledge and skills, thereby improving their level of awareness of agricultural production techniques and information accumulation. The farmers who do not productively use the Internet use it only for leisure and entertainment activities. The Internet pushes more of the same type of information according to people′s areas of interest. Those who like entertainment programs will receive more entertainment programs, and those who like production technology will receive more information about production technology. Gradually, the areas of information that people receive using the Internet will become increasingly concentrated, thereby forming an “information cocoon”. As the time spent using the Internet increases, the gap between farmers who do not use the Internet productively and those who use it productively becomes increasingly large regarding their knowledge of agricultural production technologies and their willingness and behavior to adopt new technologies. In the survey sample, 40.49% of the farmers who used the Internet productively wanted training in ecological conservation, while this was only 17.74 percent of farmers who did not use it productively.

Second, many farmers use social Internet platforms (e.g., WeChat) to watch and learn green production technologies. Furthermore, peers get to know and communicate with each other, expanding social capital through “Internet capital” and forming a heterogeneous social network that is different from the surrounding environment and embedded in social relationship networks. The interaction, demonstration, driving, and following between farmers online and offline reduces the uncertainty of green production technology adoption and increases the willingness and behavior of green production technology adoption.

Third, farmers using the Internet to engage in e-commerce have relatively few sales transaction links, low transaction costs, increasing economic returns, and increasing willingness to make productive investments. The Internet market is significantly competitive, with complete information and transparent prices, which promotes the “high quality with high price” of green agricultural products, thereby increasing farmers′ willingness and behavior to invest in green and high-quality agricultural products and production technologies. In the survey sample, 80.12% of farmers who productively use the Internet think that the quality of agricultural products is important, which is 9.56 percentage points higher than the percentage of farmers who do not productively use the Internet. It can be shown that farmers who productively use the Internet were more willing to demand green agricultural products and green production technology. Farmers with productive Internet use can directly use the Internet to obtain product market information and services, including market services for soil testing and formulated fertilization technology.

## 4. Conclusions

Soil testing and formulated fertilization technology are green production technologies in agriculture that effectively addresses the problem of the excessive and inefficient use of chemical fertilizers. Many studies have found that the use of the Internet positively influences the adoption of soil testing and formulated technology; however, they have not distinguished between the different impacts of the different uses of the Internet on the adoption of soil testing and formulated technology. By using data of 5341 professional farmers in China in 2019, we found that 19.48% of professional farmers in China still use the Internet only for communication and entertainment functions and do not use any productive agricultural service functions of the Internet, such as acquiring knowledge and technology online, searching for product market information, and engaging in agricultural e-commerce. The percentage of farmers adopting soil testing and formulated fertilization technology is 33.36% among farmers who productively use the Internet, which is 9.96 percentage points higher than the percentage of farmers who do not productively use it. The regression results obtained using Logit, Probit, LPM, IPWRA, and AIPW models are stable and consistent. The double robust model shows that the probability of adopting soil testing and formulated fertilization technology among farmers with the productive use of the Internet increased by nearly six percentage points, an 18% increase in the probability of adoption of soil testing and formulated fertilization technology. The result indicates that the productive use of the Internet can increase the adoption of soil testing and formulated fertilization technology both statistically and economically.

The Internet is a skill-biased technical change that requires users to have specific skills. Since Chinese farmers generally have low educational attainment, their use of the Internet is still lacking. In the future, even if the Internet becomes widespread among Chinese farmers, not all farmers know “how to use” the Internet. The “information divide” created by Internet use is more pronounced, not only for the adoption of green agricultural production technologies but also for economic returns to farmers. China should increase training in Internet use so that more farmers, particularly those with low skills and educational attainment, can learn to use the Internet well. Thus, the Internet can play a positive role in promoting the adoption of green production technologies in agriculture.

The shortcoming of this study is that, due to limited data availability, only the impact of the different uses of the Internet (with or without productive use) on farmers′ green production technology adoption was analyzed, and the impact of the frequency and timing of the different uses was not included. We also did not have details about the adoption of soil testing and formulated fertilization technology, which prevents us from analyzing it in-depth. Future studies should address those two data issues.

## Figures and Tables

**Table 1:** Internet use of professional farmers.

Internet Applications	No Productive Use		Productive Use (Includes Any of the Following Uses)		
Percentage (%)	18.97		81.03		
Internet Applications	Call, WeChat	Leisure, Entertainment	Learning about agricultural production and management	Obtain product market information	E-commerce of agricultural products
Percentage (%)	95.17	45.98	65.12	64.33	47.78

**Table 2:** Descriptive statistics of the main variables.

Variable Type	Variables	All Samples	No Productive Use	Productive Use	*p*-Value
		N = 5341	N = 1013	N = 4328	
	Soil formula fertilization technology adoption	31.47	23.40	33.36	<0.001
Personal Characteristics	Gender (male)	80.92	78.58	81.47	0.035
	Age (years)	45.94	48.71	45.29	<0.001
	Education level				<0.001
	*Elementary school or below*	5.00	12.24	3.30	
	*Junior high school*	51.34	56.76	50.07	
	*High school*	33.70	26.26	35.44	
	*College and above*	9.96	4.74	11.18	
	Technical skills	26.64	26.75	26.62	0.93
	Agricultural production and management training	97.34	96.35	97.57	0.029
	Village officials	22.09	20.83	22.39	0.28
Family Characteristics	Home entry road type				<0.001
	*Dirt road*	15.20	22.31	13.54	
	*Gravel road*	11.18	13.13	10.72	
	*Concrete or asphalt road*	73.62	64.56	75.74	
	Number of agricultural laborers	2.42	2.40	2.42	0.51
Production and management characteristics	Total land operation area (mu)	137.79	98.65	146.95	<0.001
	Type of production and operation (combined planting and animal husbandry)	27.93	31.00	27.22	0.016
	Main crops				<0.001
	*Wheat*	9.41	6.37	10.15	
	*Corn*	9.21	5.82	10.00	
	*Rice*	14.68	14.51	14.72	
	*Other food categories*	24.62	30.70	23.20	
	*Vegetables*	4.06	4.74	3.90	
	*Fruits*	12.34	13.23	12.13	
	*Other cash crops*	21.46	16.49	22.62	
	Family farm	25.84	20.43	27.10	<0.001
	Cooperative membership	38.94	33.46	40.23	<0.001
	Cooperation with agricultural companies	31.60	27.34	32.60	0.001

**Table 3:** The marginal effect of different variables on the adoption of soil testing and formulated fertilization technology from Logit, Probit, and LPM models.

	(1)	(2)	(3)
Variables	Logit	Probit	LPM
Productive use of the Internet	0.046 *	0.046 **	0.040 *
	0.024	0.023	0.021
Male	0.039 *	0.040 *	0.038 *
	0.021	0.021	0.021
Age	0.001	0.001	0.001
	0.001	0.001	0.001
Education level (with college and above as the reference group)			
*Elementary school or below*	−0.089 **	−0.087 **	−0.078 **
	0.040	0.039	0.038
*Junior high school*	−0.044 *	−0.045 *	−0.046 *
	0.025	0.024	0.025
*High school*	−0.027	−0.028	−0.028
	0.024	0.024	0.024
Technical skills staff	0.028	0.028	0.026
	0.022	0.021	0.022
Agricultural training	−0.060	−0.060	−0.059
	0.047	0.046	0.044
Village officials	−0.012	−0.011	−0.012
	0.019	0.019	0.020
Entry road (dirt road as the reference group)			
*Gravel road*	−0.009	−0.007	−0.007
	0.030	0.030	0.030
*Concrete road*	0.029	0.029	0.031
	0.022	0.022	0.022
Number of agricultural laborers	−0.015 **	−0.014 **	−0.014 **
	0.007	0.007	0.007
Log of land operating area	0.036 ***	0.037 ***	0.036 ***
	0.008	0.008	0.008
Combined planting and animal husbandry	−0.076 ***	−0.075 ***	−0.073 ***
	0.020	0.020	0.020
Top crops (with wheat as the reference group)			
*Corn*	−0.139 ***	−0.142 ***	−0.148 ***
	0.047	0.047	0.049
*Rice*	−0.006	−0.008	−0.011
	0.044	0.044	0.045
*Other food crops*	−0.101 **	−0.106 **	−0.107 **
	0.049	0.048	0.047
*Vegetables*	−0.097 **	−0.096 **	−0.108 **
	0.047	0.046	0.047
*Fruits*	−0.089 **	−0.090 **	−0.103 **
	0.045	0.045	0.047
*Other cash crops*	−0.143 ***	−0.143 ***	−0.149 ***
	0.041	0.041	0.041
Family farm	0.058 **	0.059 **	0.062 **
	0.024	0.023	0.024
Cooperative membership	0.003	0.001	0.002
	0.018	0.018	0.019
Cooperation with agricultural companies	−0.019	−0.019	−0.016
	0.022	0.021	0.022
Provincial dummies	Yes	Yes	Yes
N	5351	5351	5351
R^2^/pseudo R^2^	0.098	0.099	0.114

Note: * *p* < 0.1, ** *p* < 0.05, *** *p* < 0.01; Robust standard error is placed in the second row.

**Table 4:** The marginal effect of different variables on the selection of productive use of the Internet from Logit, Probit, and LPM models.

	(1)	(2)	(3)
Variables	Logit	Probit	LPM
Productive use of the Internet in the county or district	0.746 ***	0.755 ***	0.941 ***
	0.037	0.036	0.044
Broadband access	0.051 ***	0.051 ***	0.060 ***
	0.016	0.016	0.018
Male	0.027 **	0.024 *	0.021 *
	0.013	0.013	0.013
Age	−0.007 ***	−0.007 ***	−0.007 ***
	0.001	0.001	0.001
Education level (with college and above as the reference group)			
*Elementary school or below*	−0.157 ***	−0.162 ***	−0.181 ***
	0.031	0.031	0.032
*Junior high school*	−0.073 ***	−0.072 ***	−0.061 ***
	0.017	0.017	0.015
*High school*	−0.033 *	−0.033 **	−0.022
	0.017	0.017	0.015
Technical skills staff	−0.026 **	−0.026 **	−0.019 *
	0.012	0.012	0.011
Agricultural training	0.021	0.019	0.018
	0.028	0.028	0.032
Village officials	0.034 ***	0.032 ***	0.029 **
	0.012	0.011	0.012
Entry road (dirt road as the reference group)			
*Gravel road*	0.030	0.033 *	0.037 *
	0.020	0.019	0.021
*Concrete road*	0.071 ***	0.071 ***	0.074 ***
	0.015	0.014	0.015
Number of agricultural laborers	0.002	0.003	0.002
	0.005	0.005	0.005
Log of land operating area	0.023 ***	0.023 ***	0.022 ***
	0.004	0.004	0.004
Combined planting and animal husbandry	0.029 ***	0.028 ***	0.031 ***
	0.011	0.011	0.012
Top crops (with wheat as the reference group)			
*Corn*	−0.011	−0.010	0.005
	0.028	0.026	0.023
*Rice*	0.003	0.005	0.014
	0.025	0.024	0.021
*Other food crops*	−0.003	−0.002	0.014
	0.036	0.034	0.030
*Vegetables*	0.044 *	0.044 *	0.065 ***
	0.026	0.024	0.022
*Fruits*	0.052 **	0.055 **	0.074 ***
	0.024	0.023	0.019
*Other cash crops*	0.034	0.035	0.041 **
	0.025	0.024	0.021
Family farm	−0.012	−0.013	−0.012
	0.013	0.013	0.012
Cooperative membership	−0.005	−0.005	−0.003
	0.011	0.011	0.011
Cooperation with agricultural companies	0.009	0.008	0.011
	0.011	0.011	0.011
Provincial dummies	Yes	Yes	Yes
N	5341	5341	5341
R^2^/pseudo R^2^	0.235	0.237	0.227

Note: * *p* < 0.1, ** *p* < 0.05, *** *p* < 0.01; Robust standard error is placed in the second row.

**Table 5:** The predicted probability of adoption of soil testing and formulated fertilization technology from double robust models.

Variables	IPWRA Model			AIPW Model		
	Logit	Probit	LPM	Logit	Probit	LPM
No productive use	0.2588 ***	0.2582 ***	0.2575 ***	0.2601 ***	0.2603 ***	0.2603 ***
	0.0187	0.0188	0.0192	0.0195	0.0196	0.0198
Productive use	0.3210 ***	0.3207 ***	0.3207 ***	0.3210 ***	0.3210 ***	0.3208 ***
	0.0073	0.0072	0.0073	0.0074	0.0074	0.0074
ATE	0.0621 ***	0.0625 ***	0.0632 ***	0.0609 ***	0.0607 ***	0.0605 ***
	0.0198	0.0200 ***	0.0204	0.0206 ***	0.0208 ***	0.0209

Note: *** *p* < 0.01; Bootstrapped standard error with 1000 replications is placed in the second row.

## Data Availability

The data are not publicly available due to personal privacy and nonopen access to the research program.
